# Eugenol alleviates acrylamide-induced rat testicular toxicity by modulating AMPK/p-AKT/mTOR signaling pathway and blood–testis barrier remodeling

**DOI:** 10.1038/s41598-024-52259-1

**Published:** 2024-01-22

**Authors:** Dalia O. Saleh, Sara M. Baraka, Gehad A. Abdel Jaleel, Azza Hassan, Omar A. Ahmed-Farid

**Affiliations:** 1 Pharmacology Department, National Research Centre, Giza, 12622 Egypt; 2 Chemistry of Natural Compounds Department, National Research Centre, Giza, 12622 Egypt; 3 Pathology Department, Faculty of Veterinary Medicine, Cairo University, Giza, Egypt; 4 Physiology Department, National Organization for Drug Control and Research, Giza, Egypt

**Keywords:** Biochemistry, Biomarkers, Diseases, Health care, Medical research

## Abstract

This study aimed to investigate the effects of eugenol treatment on reproductive parameters in acrylamide (ACR)-intoxicated rats. The study evaluated alterations in relative testes and epididymides weights, sperm quality, serum hormonal status, seminal plasma amino acids, testicular cell energy and phospholipids content, oxidative and nitrosative stress parameters, adenosine monophosphate-activated protein kinase/ phosphoinositide 3-kinase/phosphor-protein kinase B/mammalian target of rapamycin (AMPK/PI3K/p-AKT/mTOR) signaling pathway, blood–testis barrier (BTB) remodeling markers, testicular autophagy and apoptotic markers, as well as histopathological alterations in testicular tissues. The results revealed that eugenol treatment demonstrated a significant improvement in sperm quality parameters, with increased sperm cell concentration, progressive motility live sperm, and a reduction in abnormal sperm, compared to the ACR-intoxicated group. Furthermore, eugenol administration increased the levels of seminal plasma amino acids in a dose-dependent manner. In addition, eugenol treatment dose-dependently improved testicular oxidative/nitrosative stress biomarkers by increasing oxidized and reduced glutathione levels and reducing malondialdehyde and nitric oxide contents as compared to ACRgroup. However, eugenol treatment at a high dose restored the expression of AMPK, PI3K, and mTOR genes, to levels comparable to the control group, while significantly increasing p-AKT content compared to the ACRgroup. In conclusion, the obtained findings suggest the potential of eugenol as a therapeutic agent in mitigating ACR-induced detrimental effects on the male reproductive system via amelioration of ROS-mediated autophagy, apoptosis, AMPK/p-AKT/mTOR signaling pathways and BTB remodeling.

## Introduction

Acrylamide (ACR) is reactive water soluble α, β-unsaturated carbonyl monomer, extensively used in many fields such as industry of polymeric products and even laboratory personal work^1^. Besides to these sources of exposure that mostly restricted to certain occupations, cigarette smoking liberates a high ACR ratio^2^. An issue of significant global concern is the presence of ACR, which serves as a major by-product during thermal cooking process of starchy foods at high temperature representing a primary source of human exposure of ACRto about 1 μg/kg bw/day^3^. Due to its water soluble characteristics and low molecular weight, ACRis readily absorbed into the body via inhalation, skin and gastrointestinal tract resulting in neurotoxicity, genotoxicity, hepato-renal toxicity and reproductive organs toxicity^4^. As per the World Health Organization (WHO), the typical intake of ACR through diet is calculated to be around 1 µg/kg body weight/day, rising to 4 µg/kg body weight/day for individuals with high consumption^5^.

Numerous human and animal researches have been conducted to elucidate the exact underlying mechanism of ACR toxicity. Accumulative evidences reported that ACR toxicity occurs mainly via oxidative stress insult^6,7^. Furthermore, in vitro studies have reported that ACR mediated oxidative stress evidenced by creation of Michael-type adduct with cysteine-containing glutathione (GSH) with a subsequent increase in reactive oxygen species (ROS) production^8,9^. By the action of CYP2E1, ACR is metabolized to more reactive metabolite; glycidamide (GA) that conjugates with GSH resulting in its depletion with more ROS production exacerbating oxidative stress process^10^.

Among the numerous mentioned toxic effects of ACR, testicular toxicity is paid a great attention as a public health concern. Several investigations have been conducted to investigate the exact mechanism of ACR-induced testicular toxicity in rodents^11,12^. Testis has been suggested to be the target organ for the detrimental effects of ACR, particularly targeting Sertoli and Leydig cells^13^. In the process of spermatogenesis, ACR could induce genotoxicity either directly on DNA or indirectly by impairing the DNA repair function of Sertoli cells. Alternatively, ACR might influence reproductive processes by disrupting the balance of sex hormones. Notably, disturbances in endocrine function have been observed in animals treated with ACR, particularly in males^14,15^.

Alterations in gonadal and pituitary hormones have been recorded and found to be associated with histopathological alterations and spermatogenesis dys-regulation but no clear mechanism have been delineated. Thus, it is important exploring bioactive compounds that can combat the detrimental effects of oxidative stress followed ACR exposure.

Eugenol (4-allyl-2-methoxyphenol), the main component of clove (*Syzygium aromaticum* L. Merr. & L. M. Perry), is a phenolic compound from the class of phenylpropanoids^16^. It is widely utilized in the food industry as a preservative agent due to its antioxidant and antimicrobial properties, and as a natural food flavoring agent, as well as in the packaging materials development^17^. Moreover, it is highly utilized for therapeutic applications as antiseptic, anti-inflammatory, antibacterial, antiviral, analgesic and antioxidant agent^18–21^. It acts as a free radical scavenger by diminished ROS generation and enhancing the intracellular antioxidant defense mechanisms. Hence, several reports have investigated the effect of eugenol in the treatment of neurodegenerative diseases, hypercholesterolemia, diabetes, hypertension, inflammatory diseases, and cancer^21–24^.

Eugenol has captivated the interest of researchers due to its functional attributes. However, the possible protective role/impact of eugenol on the ACR-induced testicular toxicity has not yet been previously addressed. Thus, the current study aimed at investigating the potential of eugenol to attenuate ACR-induced testicular impairment and spermatogenesis disruption. Notably, the underlying molecular mechanisms of eugenol were speculated, specifically those related to autophagy, apoptosis, and oxidative stress including the adenosine monophosphate-activated protein kinase/ phosphoinositide 3-kinase/phosphor-protein kinase B/mammalian target of rapamycin (AMPK/PI3K/p-AKT/mTOR) pathways and blood–testis barrier (BTB) disorder.

## Materials and methods

### Animals

In the present study, four-month-old male Wistar Albino rats (n = 32) weighing 180–220 g were used. The animals were procured from the National Research Centre’s animal house (Giza, Egypt). During the study, the rats were housed under appropriate conditions in a room with 12 h of daylight; suitable cages, fed standard rat pellet chow obtained from the animal house at the National Research Centre, and allowed to have free access to water via dropper-tipped bottles.

### Ethics

The experiment was conducted in adherence to the ethical guidelines for routine experimental animal studies set forth by the National Research Centre's Medical Research Ethics Committee (MREC). The committee approved the experimental protocol, ensuring its alignment with the directives outlined in the Guide for Care and Use of Laboratory Animals (US-NIH Publication No. 24410112022) and the ARRIVE guidelines.

### Drugs and chemicals

Acrylamide (ACR) was purchased from Sigma-Aldrich (A3553), St. Louis, MD, USA, freshly prepared in dis. water and the concentration was adjusted at 0.5 mL/200 g rat bw. Eugenol was acquired from Sigma-Aldrich (E51791-99%), St. Louis, MD, USA. It was prepared in 1 mL olive oil to give a concentration of 0.2 mL/200 g rat bw. All other chemicals were of the utmost analytical quality available.

### In vivo study

#### Experimental design

A total of thirty-two male Wistar rats were equally divided into four groups; control group in which rats received the vehicles; 1 mL of olive oil/kg and 2.5 mL dis. water; model group (ACR) in which rats received oral administration of ACR (20 mg/kg bw) once daily for 28 days^25^ and 1 mL/kg olive oil (vehicle of eugenol). Eugenol treated groups; Eu50 + ACR or Eu100 + ACR group; in which animals were treated orally with eugenol (50 or 100 mg/kg bw/day, respectively)^26^ along with a daily oral dose of ACR (20 mg/kg bw) for 28 days. Eugenol was given before ACR exposure 1 h interval.

Twenty-four hours after the last dose of drugs, the rats were weighed then the blood samples were collected from the retro-orbital sinus under Ketamine–Xylazine anesthesia (50–5 mg/kg, i.p)^27^. Sera samples were separated and stored at – 80 °C. Rats were sacrificed by decapitation; testes and epididymides were collected and weighed to calculate their relative weights. Portions of tissues were either kept at − 80 °C for further biochemical analyses, while other parts of tissues were fixed in 10% formalin for the histopathological and immunohistochemical investigations.

#### Samples preparation

The epididymides and testes were precisely separated. Each epididymal caudal was pulverized then semen was collected in 1 mL of phosphate buffer saline (PBS, pH = 7.4). Sperm cell suspension was take 1:200 PBS pH 7.4. Subsequently, the processed semen samples were subjected to centrifugation at 4 °C and 600×*g* for duration of 20 min. This procedure facilitated the isolation of seminal plasma supernatant, which was then preserved at − 20 °C prior to the assay. A 10% testicular homogenate (1 g of testicular tissue was homogenized in 10 mL buffer) was meticulously prepared in 0.05 M PBS (pH 7), utilizing a polytron homogenizer. The resulting homogenate was subjected to centrifugation at 10,000 rpm for a span of 20 min, effectively eliminating cell debris, unbroken cells, nuclei, erythrocytes, and mitochondria. The ensuing supernatant (Cytosolic fraction) was carefully separated for further analysis and stored at − 80 °C until the assay. For production of nuclear fraction, the remaining pellet was immersed in 500 μL Nuclear Lysis Buffer. The suspension was then spun for 10 min at 14,000 rpm in a cooling centrifuge and the acquired supernatant (nuclear fraction) was collected for other biochemical assessment.

#### The relative testes or epididymides weight

The relative testes or epididymides weight was calculated as follows: relative organ weight (%) = [organ weigh (g)/final body weight (g)] * 100.

### Sperm examination

#### Sperm cell concentration

To calculate the sperm cell concentration (SCC), the sperm suspension samples were diluted to 500 μL using formaldehyde fixative (10% formalin in PBS). A volume of 10 μL of the resultant solution was placed in a haemocytometer and let to stand for 7 min to calculate the number of the settled sperms/250 small squares of the haemocytometer. Then the number of spermatozoa (million/ mL) was counted as sperm cell concentration (SCC)^28,29^.

#### Percentage of progressive and individual motility of spermatozoa

After each collection, one drop of fresh semen was examined on a glass slide at 37 °C, two drops of warm 0.9% NaCl were added, and a cover slip was immediately utilized. The examination was performed at a high magnification (400×) to compute the sperm progressive (PM) and individual motility (IM) percentages.

#### Percentage of living and abnormal spermatozoa

To evaluate sperm vitality, a volume of 40 μL of liquefied semen was thoroughly mixed with 10 μL of eosin Y (1 c/o), and one drop of the prepared mixture was placed to a clean slide to determine the number of viable and abnormal sperm under a light microscope^30^. At least two hundred sperms were counted and examined; the sperms that stained pink or red were represented as dead, while the sperms with no stain were reflected the viable sperms, and the percentage of viable sperm was computed. The percentage of abnormally shaped sperms that appeared morphologically defects in tail and head was estimated.

### Biochemical studies

#### Determination of serum hormonal profile

The serum levels of total testosterone (Cusabio, Houston, Cat. No. CSB-E0597r, TX, USA), follicle-stimulating hormone (FSH, Abnova, Cat. No. KA2330, USA) and luteinizing hormone (LH, Cat. No. NBP2-61257, Novus Biologicals™, USA) levels were measured using ELISA (Enzyme Linked Immunosorbant Assay) kits. The analyses were performed in accordance with manufacturer’s instruction.

#### Determination of seminal plasma essential and non- essential amino acids

Using the high performance liquid chromatography (HPLC, Agilent HP 1200 series), the levels of amino acids in seminal plasma were determined^31^. Briefly, the seminal plasma samples underwent centrifugation, and a 400 μL volume of the resulting supernatant was concentrated under reduced pressure. Subsequently, it was suspended in a 100 μL coupling buffer comprised of acetonitrile, ethanol, tri-ethylamine, and water in a ratio of 10:5:2:3, followed by re-evaporation under reduced pressure. The mobile phase utilized a seven-step gradient, with increasing concentrations of solvent B ranging from 5 to 70%. Solvent A consisted of 50 mM ammonium acetate (pH 6.5), while solvent B was composed of a mixture of 100 mM ammonium acetate (pH 6.5) and acetonitrile in a 1:1 ratio. The flow rate was maintained at 2 mL/min, and the temperature was set to 500 °C. The samples were re-suspended in a coupling buffer (90 μL) along with phenyl isothiocyanate (PITC) (10 μL) and then incubated for 5 min at room temperature. Subsequently, they were evaporated and re-suspended in a solution containing 240 μL of 50 mM ammonium acetate buffer and 10 μL of methanol at pH 7. The separation of amino acids was achieved using an ultrasphere C18 reversed phase column (Nucleosil^®^ 5 μm C18 100 Å, LC Column 250 × 4.6 mm, Ea) with a wavelength of 254 nm and a UV detector.

#### Determination of testicular tissue ATP, ADP and AMP contents (μg/g tissue) by HPLC

The separation of testicular tissue ATP (adenosine triphosphate), ADP (adenosine diphosphate) and AMP (adenosine monophosphate) was conducted using HPLC (Agilent HP 1200 series, USA). The Ultrasphere ODS EC 250 × 4.6 mm column (Catalog Number: HI235329) served as the analytical column. The ATP, ADP, and AMP contents (expressed in μg/g tissue) within the samples were determined by comparing them with standards acquired from Sigma Aldrich. The final report and chromatograms were generated using the ChemStation program, utilizing a wavelength of 254 nm for detection^32^. Whereas, the charge cell energy or total adenylate energy charge (AEC) was evaluated according to the method of Pradet and Raymond^33^ as follows:

$${\text{AEC}}\, = \,\left( {{\text{ATP}}\, + \,0.{\text{5 ADP}}} \right)/\left( {{\text{ATP}}\, + \,{\text{ADP}}\, + \,{\text{AMP}}} \right).$$

#### Determination of testicular tissue phosphatidylserine (PS) and phosphatidylcholine (PC) contents by HPLC

##### Phospholipids extraction

Total phospholipids were extracted as described by Folch et al. with minor modification^34^. In brief, 1 g of acacia gum suspension acquired from Abhishek Impex India (Gum Arabic Tree-500 g) was gently relocated to a graduated glass tube. A mixture of chloroform: methanol (2v:1v) was added to the glass tube at twice volume as that of acacia extract volume. The suspension was vigorously agitated and subjected to centrifugation at 2500*g* for duration of 10 min. Following centrifugation, the supernatant was carefully removed. A methanol: water solution (in a 1:1 volume ratio) was subsequently introduced to the remaining supernatant, constituting a quarter of its original volume. This mixture was thoroughly mixed and subjected to another round of centrifugation at 2500*g* for 10 min. The resulting supernatant and the boundary layer were then discarded. The residual supernatant was subsequently transferred to a separate glass tube, where it was subjected to a stream of nitrogen to facilitate drying. The dried extract was stored at a temperature of – 20 °C. Prior to high-performance liquid chromatography (HPLC) analysis, the extracted phospholipid was reconstituted using a mobile phase solvent containing 20% chloroform.

##### HPLC chromatographic separation

The separation of different phospholipids was achieved using an isocratic high-performance liquid chromatography (HPLC) system, specifically the Agilent 1200 Series, which was equipped with a computerized solvent delivery system and a UV detector. This setup was located in Santa Clara, CA, USA. A porasil silica gel column with a particle size of 10 µm was employed for the chromatographic separation. For the HPLC analysis, 20 µL of samples were injected. The elution was carried out utilizing a degassed mobile phase composed of acetonitrile, methanol, and 85% phosphoric acid in a ratio of 96:3:1 (v/v/v). The mobile phase was delivered at a flow rate of 0.80 mL/min. Detection of the effluent was performed at a wavelength of 203 nm using a UV detector. The concentration of each sample was determined by comparing it with corresponding phospholipid standards. The phospholipid standards used were PS (phosphatidylserine) and PC (phosphatidylcholine), which were procured from Sigma Chemical Company located in St. Louis, MO, USA. Prior to analysis, each standard was prepared in a concentration of 1 mg/mL using a mixture of chloroform and methanol in a 2:1 (v/v) ratio. These standards were stored at a temperature of – 20 °C. It is worth noting that all chemicals used in this process were of analytical-reagent grade.

#### Determination of the testicular tissue oxidative and nitrosative stress parameters using HPLC

The thiol compounds of oxidized and reduced glutathione (GSSG and GSH) were analyzed using High-Performance Liquid Chromatography (HPLC) with an Agilent HP 1200 series system from the USA. This HPLC system was equipped with a quaternary pump, a column oven, a Rheodine injector with a 20 μL loop, and a UV variable wavelength detector. The analytical column employed was a μBondapak C18 Column with a particle size of 10 μm and a pore size of 125 Å. Its dimensions were 3.9 mm × 300 mm (Catalog Number: WAT027324). To quantify the glutathione (both oxidized and reduced forms), reference standards were utilized. These standards were obtained from Sigma Chemical Co. and served as a basis for comparison with the samples. The results of the analysis were expressed in terms of micromoles per gram of tissue (μmol/g tissue)^35,36^.

To determine malondialdehyde (MDA) levels, the samples underwent analysis using an HPLC apparatus. The analytical column employed was a Supelcosil C18 column with a particle size of 5 μm and a pore size of 120 Å. Its dimensions were 250 × 4.6 mm (Catalog Number: 58298). For the preparation of the MDA standard, 25 μL of 1,1,3,3-tetraethoxypropane (TEP) was dissolved in 100 mL of water, resulting in a 1 mM stock solution. A working standard was created by hydrolyzing 1 mL of the TEP stock solution in 50 mL of 1% sulfuric acid and incubating it for 2 h at room temperature. This working standard was then further diluted by adding 1% sulfuric acid to achieve a concentration of 20 nmol/mL TEP, resulting in a final concentration of 1.25 nmol/mL for use as a standard for total MDA estimation^37^.

The determination of nitric oxide (NO) level, specifically nitrate/nitrite, was carried out using the Agilent HP 1200 seriesHPLC apparatus from the USA, following the previously described procedure. The analytical column employed was an anion exchange PRP-X100 Hamilton column with dimensions of mm and a particle size of 10 μm. The mobile phase consisted of a mixture of 0.1 M NaCl and methanol, with a volume ratio of 45:55. The flow rate was set at 2 mL/min, and the wavelength of detection was adjusted to 230 nm. This configuration allowed for the analysis of nitric oxide levels in the form of nitrate/nitrite^38^. The obtained chromatogram enabled the determination of the concentration within the sample by comparing it to the standard obtained from Sigma Aldrich.

#### Determination of testicular MMP-9, CX-43, P-AKT, p-AMPK, 17β-HSD, nuclear Nrf2, Beclin-1 and Caspase-3 levels

Rat Matrix metallopeptidase 9 (MMP-9, Cloud-Clone Corp, Cat. No. SEA553Ra, TX, USA), Connexin 43 (CX-43, Fine Test, Cat. No. ER0881, Wuhan, China), phosphorylated Akt (p-Akt, MyBioSource, Inc, Cat. No. MBS1600201, CA, USA), p-AMPK (Fine Test^®^, Cat. No. ER0730, Wuhan, Hubei, China), 17 β-Hydroxysteroid Dehydrogenase type 3 (17β-HSD3, Biomatik Corporation, Cat. No. EKU10595, Kitchener, Ontario, Canada), the autophagic marker; Beclin-1 (BIOMATIK, Cat. No. EKU026858, USA) as well as the apoptotic marker; Caspase-3 (Cloud-Clone Corp, Cat. No. HEA626Ra, TX, USA) levels were assessed in testicular homogenates (Cytoplasmic fraction) using ELISA kits in accordance with manufacturers’ instructions.

Meanwhile, the nuclear fraction was used to evaluate the nuclear content of nuclear factor erythroid 2 (Nrf2), using a rat ELIZA kit (Cat. No. NBP3-08161, NovusBiologicals, LLC, 10771E, EasterAve, Centennial, CO 80112, USA) following the manufacturers’ outlines. The protein content within the tissue was assessed following the protocol outlined by Bradford, utilizing the protein estimation kit from Genei, Bangalore^39^.

### Molecular studies

#### Quantitative mRNA expression analysis by real-time PCR (qRT-PCR)

The total cellular RNA was purified from testicular tissue using Direct-zol RNA Miniprep Plus kit (Cat# R2072, ZYMO RESEARCH CORP. USA) following the instructions provided. The concentration and purity of the isolated RNA were evaluated by 260/280 nm UV absorbance ratios. The Invitrogen™ SuperScript™ IV One-Step RT-PCR System (Cat# 12594100, Thermo Fisher Scientific, Waltham, MA USA) was utilized for reverse transcription of extracted RNA followed by PCR in one step. The synthesis of first-strand cDNA was accomplished through reverse transcription, employing an oligo-(dT) primer and the MML V first-chain synthesis kit in accordance with the instructions provided by the manufacturer, Invitrogen. The expression level of testicular AMPK, PI3K and mTOR genes were analysed in a Real-Time PCR System (Applied Biosystems, U.S.A.) using SYBR Green Mix (Invitrogen) and the following primers for AMPK, forward: 5′-AGCTCGCAGTGGCTTATCAT-3′, reverse: 5′-GGGGCTGTCTGCTATGAGAG-3′ (NM_023991.1); PI3K, forward: 5′-TTAAACGCGAAGGCAACGA-3′, reverse: 5′-CAGTCTCCTCCTGCTGTCGAT-3′′ (XM_032898971.1); mTOR, forward: 5′-ACGAAGGAGACAGACCGAAG-3′, reverse: 5′-CGACGAAGTCACTAGATTCA-3′ (AM_943028.1); Cholesterol side-chain cleavage enzyme (CYP11A), forward: 5′-TCCCATGGCTACAGGTCTTC-3′, reverse: 5′-CTGCTTTAGGAGACGCAGGT-3′ (XM_014154002.1) and GAPDH, forward: 5′-CCTCGTCTCATAGACAAGATGGT-3′, reverse: 5′-GGGTAGAGTCATACTGGAACATG-3′ (NM_001394060.2). The thermal profile cycling condition was as follows: 55 °C for 10 min, 95 °C for 2 min, followed by 40 cycles of 95 °C for 10 s, 55 °C for 10 s and 72 °C for 30 s then final extension (1cycle) at 72 °C for 5 min. After the RT-PCR run the data were expressed in Cycle threshold (Ct). The PCR data sheet includes Ct values of assessed gene (*PI3K, AMPK and mTOR*) versus the corresponding housekeeping gene (*GAPDH*). The relative quantification (RQ) of each target gene is quantified and normalized to the housekeeping gene according to 2^−ΔΔCt^ method.

### Histopathological examination

The testes from all groups were removed and fixed in Bouin solution for 48 h. The tissues were processed and embedded in paraffin blocks. After that, the tissues were cut into 4 µm-thick sections and stained with hematoxylin and eosin for light microscopical examination. For assessment of testicular injury, the tissues were semiquantitively assessed in ten random low-power fields (10×) according to the number of seminiferous tubules affected, as described by Lanning et al.^40^ with some modifications. A five-grading system is used in this assessment, in which Grade 1 (minimal) contains < 10% of the tubules, Grade 2 (slight) contains 11–25%, Grade 3 (moderate) contains 26–50%, Grade 4 (marked) contains 51–75%, and Grade 5 (severe) contains 76–100%.

### Immunohistochemical analysis

Caspase-3 immunohistochemical staining for assessment of apoptosis in the testes and PCNA immunohistochemical staining for determination of the proportion of proliferating cells in the testis were carried out. Briefly, the paraffin-embedded tissues were sectioned, dewaxed, and rehydrated in xylene. Antigen retrieval was then carried out in a microwave. After that, the sections were treated with 3% hydrogen peroxide to inhibit endogenous peroxidase activity. The tissues were then incubated with rabbit monoclonal anti-caspase-3 [EPR18297] (ab184787) (Abcam) and mouse monoclonal anti-PCNA [PC10] (ab29) (abcam) as primary antibodies. Following washing with phosphate buffered saline and secondary antibody incubation, diaminobenzidine was added to stain the sections. The sections were then counterstained with hematoxylin. Caspase-3 and PCNA were semiquantitively assessed in ten random high-power fields (40×), according to the percentage of positive cells, in which scale 0 = no staining, scale 1 = denotes staining in 25%, scale 2 = 25–50%, scale 3 = 51–70%, and scale 4 > 70%.

### Statistical analysis

All the data are presented as mean ± SEM and the Shapiro–Wilk test used for normality check at p > 0.05^41^. One-way analysis of variance (ANOVA) followed by Tukey’s post hoc test were applied for multiple comparison, where values are significantly different at p < 0.05. All the statistical studies were implemented using Statistical Package for Social Science (SPSS, version 17.0, Inc., Chicago, USA).

## Results

### Changes in relative testes or epididymides weight

A remarkable decline in both relative testes and epididymides weight has been noticed in ACR-intoxicated rats, compared to control group. By contrast, eugenol treated groups showed a significant increase in the relative testes and epididymides weight compared to ACR-group (Table 1).

**Table 1:** Effect of eugenol treatment on relative testes and epididymides weight (%) in ACR-induced testicular toxicity in rats.

Groups	Relative weight (%)	
	Testes	Epididymides
Control	2.17 ± 0.13^a^	0.36 ± 0.01^a^
ACR	1.90 ± 0.03^b^	0.31 ± 0.01^b^
Eu50 + ACR	2.22 ± 0.10^a^	0.36 ± 0.17^a^
Eu100 + ACR	2.33 ± 0.10^a^	0.39 ± 0.02^a^

Data are expressed as mean ± SEM (n = 6–8).

Values with dissimilar letters are significantly different at p < 0.001.

### Effect of eugenol treatment on sperm quality

**Figure 1:**
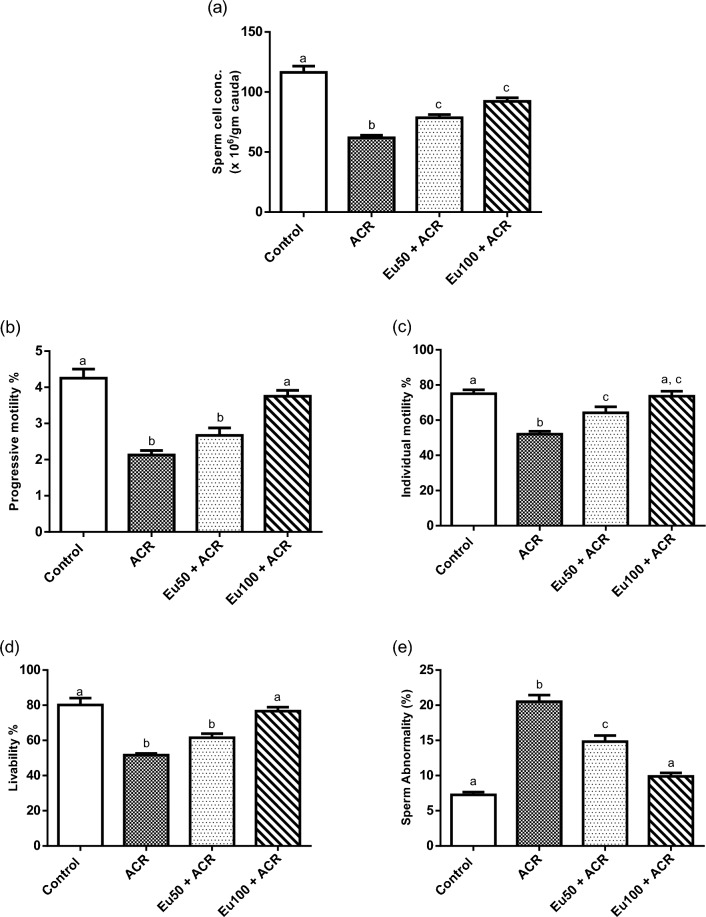
Effect of eugenol treatment on sperm quality, in terms of sperm cell concentration (**a**), and mass motility; percentage of progressive and individual motility (**b**,**c**, respectively), percentage of living normal spermatozoa (**d**) and abnormal spermatozoa (**e**) in ACR-induced testicular toxicity in rats. Data are expressed as mean ± SEM (n = 6). Columns with dissimilar letters are significantly different at p < 0.05.

Concerning SCC and percentage of PM, IM and live sperm, ACR exposure significantly reduced all these parameters along with a marked increase in percentage of abnormal sperm comparing to the control rats. On the other hand, a marked improvement was observed in semen quality parameters as manifested by increase in SCC, %PM, %LM and %live sperm, and reduction in % abnormal sperm, in eugenol treated groups in a dose dependent level when compared to ACR-intoxicated group (Fig. 1).

### Biochemical analyses

#### Effect of eugenol treatment on serum hormonal status

**Figure 2:**
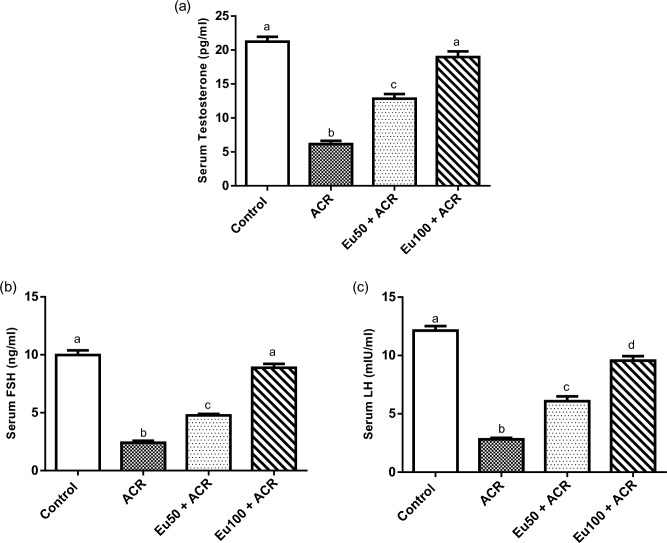
Effect of eugenol treatment on serum testosterone (**a**), FSH (**b**) and LH (**c**) in ACR-induced testicular toxicity in rats. Data are expressed as mean ± SEM (n = 8). Columns with dissimilar letters are significantly different at p < 0.05.

A significant reduction in serum testosterone (71%), FSH (75.95%) and LH (76.83%) levels has been observed in ACR-intoxicated group, compared to the control rats. Oral administration of eugenol has led to a marked elevation in these hormones dose dependently compared to ACR-exposed rats (Fig. 2).

#### Effect of eugenol treatment on seminal plasma essential and non-essential amino acids

As presented in Table 2, a prominent reduction in both seminal plasma essential and non-essential amino acids in ACR-intoxicated group when compared to the control rats. On the other hand, eugenol administration elevated all these amino acids dose dependently as compared to ACR-group.

**Table 2:** Effect of eugenol treatment on seminal plasma essential and non-essential amino acids in ACR-induced testicular toxicity in rats.

Parameters	Groups			
	Control	ACR	Eu50 + ACR	Eu100 + ACR
Essential amino acids (μmol/ml)				
ARG	4.56 ± 0.08^a^	3.85 ± 0.02^b^	4.00 ± 0.15^b,c^	4.32 ± 0.09^a,c^
HIS	1.61 ± 0.03^a^	1.30 ± 0.02^b^	1.57 ± 0.04^a^	1.57 ± 0.02^a^
LEU	2.70 ± 0.03^a^	2.27 ± 0.02^b^	2.48 ± 0.09^c^	2.70 ± 0.06^a^
ISOLEU	3.25 ± 0.09^a^	2.69 ± 0.05^b^	3.05 ± 0.10^a^	3.08 ± 0.06^a^
LYS	3.09 ± 0.06^a,c^	2.74 ± 0.03^b^	2.92 ± 0.05^b,c^	3.19 ± 0.09^a^
METH	1.03 ± 0.01^a^	0.89 ± 0.01^b^	1.03 ± 0.03^a^	1.07 ± 0.02^a^
VAL	2.65 ± 0.03^a^	2.26 ± 0.03^b^	2.64 ± 0.05^a^	2.63 ± 0.04^a^
THR	1.14 ± 0.03^a^	0.95 ± 0.01^b^	1.07 ± 0.02^a^	1.05 ± 0.01^a^
PHE	1.22 ± 0.03^a^	0.75 ± 0.02^b^	0.89 ± 0.01^c^	0.99 ± 0.01^d^
TRYP	0.72 ± 0.01^a^	0.66 ± 0.01^b^	0.69 ± 0.02^a,b^	0.70 ± 0.01^a,b^
Non-essential amino acids(μmol/ml)				
ASP	1.96 ± 0.04^a^	1.19 ± 0.02^b^	1.45 ± 0.06^c^	1.63 ± 0.03^d^
SER	4.09 ± 0.02^a^	2.47 ± 0.07^b^	2.93 ± 0.04^c^	3.27 ± 0.05^d^
PRO	1.04 ± 0.95^a^	0.95 ± 0.01^b^	1.03 ± 0.02^a,b^	1.01 ± 0.03^a,b^
CYS	0.81 ± 0.02^a^	0.49 ± 0.02^b^	0.57 ± 0.02^c^	0.66 ± 0.01^d^
TYR	0.98 ± 0.02^a^	0.62 ± 0.01^b^	0.79 ± 0.02^c^	0.79 ± 0.01^c^
ALA	1.14 ± 0.03^a^	0.88 ± 0.01^b^	1.00 ± 0.01^c^	1.04 ± 0.03^a,c^
GLU	4.99 ± 0.14^a^	2.92 ± 0.05^b^	3.93 ± 0.16 ^c^	4.28 ± 0.11^c^
GLY	5.13 ± 0.04^a^	4.29 ± 0.08^b^	5.03 ± 0.08^a^	5.29 ± 0.08^a^

Data are expressed as mean ± SEM (n = 6–8).

Values with dissimilar letters are significantly different at p < 0.001.

#### Effect of eugenol treatment on testicular cell energy and phospholipid content

Oral administration of ACR showed a dramatic decline in testicular tissue cellular energy as delineated by the decrease in ATP and AEC along with increase in ADP, AMP and AMP/ATP ratio, compared to the normal control group. By contrast, eugenol treatment at dose 100 mg/kg bw improved testicular cell energy as indicated by reducing ATP depletion, ADP, AMP and AMP/ATP ratio resulted in elevating AEC, in comparable with ACR-intoxicated group. Furthermore, a marked disturbance in testicular cell phospholipid content has been evidenced as indicated by elevation of PS content along with reduction in PC content in ACR-administered group, compared to the control rats. However, oral administration of eugenol at high dose significantly normalized the testicular PS and PC contents (Table 3).

**Table 3:** Effect of eugenol treatment on testicular cell energy (ATP, ADP and AMP) and phospholipid content in ACR-induced testicular toxicity in rats.

Parameters	Groups			
	Control	ACR	Eu50 + ACR	Eu100 + ACR
ATP (μg/g tissue)	86.49 ± 3.44^a^	53.70 ± 1.11^b^	60.55 ± 0.69^b^	70.55 ± 1.77^c^
ADP (μg/g tissue)	21.21 ± 0.96^a^	35.16 ± 1.27^b^	25.32 ± 1.57^a^	21.24 ± 0.75^a^
AMP(μg/g tissue)	8.86 ± 0.65^a^	14.15 ± 0.51^b^	10.58 ± 0.74^a^	9.07 ± 0.55^a^
AMP/ATP ratio	0.10 ± 0.005^a^	0.26 ± 0.009^b^	0.17 ± 0.01^c^	0.13 ± 0.008^a^
AEC	0.83 ± 0.005^a^	0.69 ± 0.003^b^	0.79 ± 0.016^c^	0.80 ± 0.006^a,c^
PS (μg/g tissue)	130.00 ± 6.11^a^	239.75 ± 7.96^b^	172.17 ± 14.18^c^	131.87 ± 7.19^a^
PC (μg/g tissue)	296.87 ± 12.07^a^	179.87 ± 2.43^b^	196.50 ± 3.04^b^	247.25 ± 8.80^a^

Data are expressed as mean ± SEM (n = 6–8).

Values with dissimilar letters are significantly different at p < 0.001.

#### Effect of eugenol treatment on testicular tissue oxidative and nitrosative stress parameters

As shown in Table 4, a remarkable imbalance in testicular antioxidant/oxidant status has been observed in ACR-intoxicated group as manifested by a significant reduction in GSH (92.08%) and GSSG (42.86%) levels along with boosting in MDA (78.97%) and NO (50%) levels, compared to the control rats. By contrast, eugenol treatment in a dose dependent-manner significantly improved testicular oxidative/nitrosative stress biomarkers as evidenced by increasing GSH and GSSG levels along with lessening MDA and NO contents, as compared to ACR-group.

**Table 4:** Effect of eugenol treatment on oxidative status (MDA, GSH, GSSG and NO) in ACR-induced testicular toxicity in rats.

Groups	MDA (nmol/g tissue)	GSH (μmol/g tissue)	GSSG (μmol/g tissue)	NO (μmol/g tissue)
Control	11.6 ± 0.42^a^	2.02 ± 0.07^a^	0.21 ± 0.005^a^	0.22 ± 0.005^a^
ACR	20.76 ± 0.31^b^	0.16 ± 0.01^b^	0.12 ± 0.003^b^	0.33 ± 0.004^b^
Eu50 + ACR	16.83 ± 0.44^c^	1.43 ± 0.04^c^	0.14 ± 0.004^c^	0.27 ± 0.007^c^
Eu100 + ACR	14.67 ± 0.09^d^	1.62 ± 0.04^d^	0.17 ± 0.003^d^	0.24 ± 0.006^d^

Data are expressed as mean ± SEM (n = 6–8).

Values with dissimilar letters are significantly different at p < 0.001.

#### Effect of eugenol treatment on testicular AMPK/PI3K/p-AKT/mTOR and Nrf2-signaling pathways

**Figure 3:**
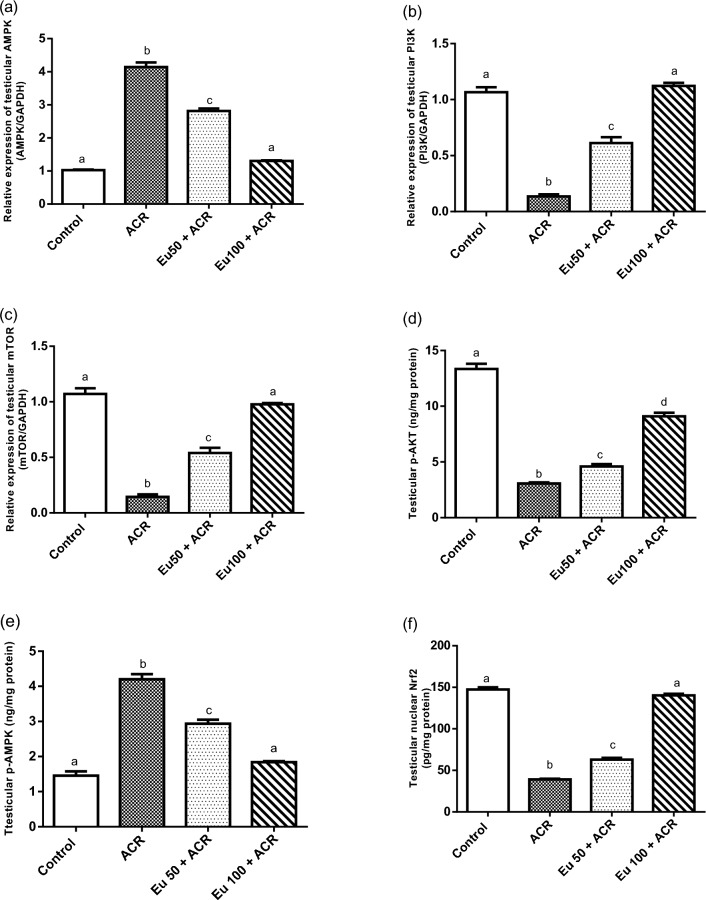
Effect of eugenol treatment on testicular AMPK (**a**), PI3K (**b**) and mTOR (**c**) gene expression, and testicular p-AKT content (**d**), p-AMPK (**e**) and nuclear Nrf2 (**f**) in ACR-induced testicular toxicity in rats. Data are expressed as mean ± SEM (n = 6). Columns with dissimilar letters are significantly different at p < 0.05.

Acrylamide administration was associated with a significant upregulation of testicular AMPK gene expression and its phosphorylated protein; p-AMPK resulted in downregulation of testicular mRNA level of PI3K and mTOR genes, and p-AKT content, compared to the control group. Meanwhile, eugenol treatment at the high dose significantly restored the testicular AMPK, p-AMPK, PI3K and mTOR genes to normal values. Additionally, a marked improvement in p-AKT level was observed in eugenol treated groups, compared to ACR-intoxicated rats. Furthermore, a significant repression in nuclear Nrf2 content was reported in ACR-challenged rats, in respect to the control group. Conversely, co-current treatment with eugenol to ACR-intoxicated rats markedly improved the testicular level of nuclear Nrf2 dose dependently as compared to the ACR-model group (Fig. 3).

#### Effect of eugenol treatment on blood–testis barrier remodeling markers

**Figure 4:**
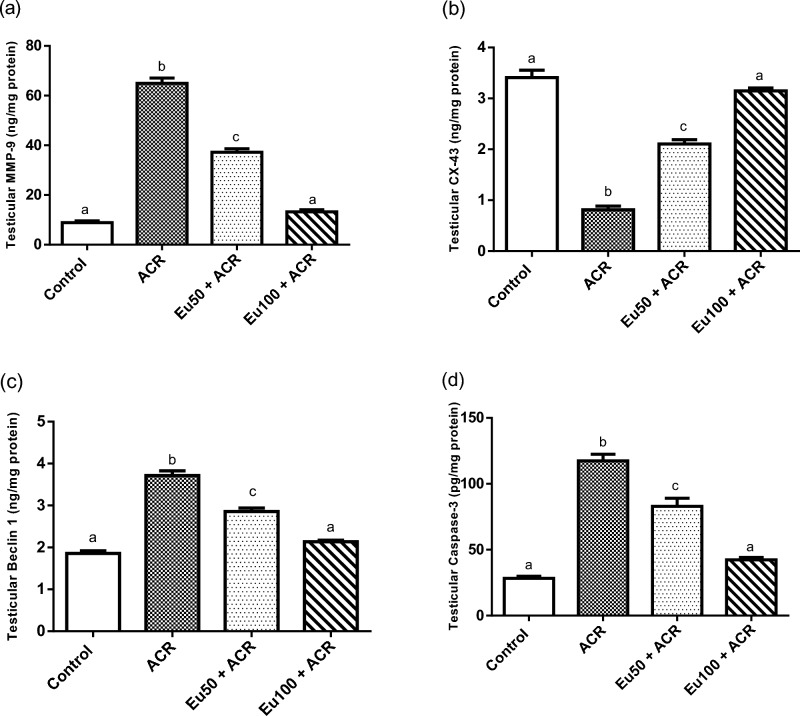
Effect of eugenol treatment on testicular MMP-9 (**a**), CX-43 (**b**), Beclin 1 (**c**) and Caspase-3 (**d**) levels in ACR-induced testicular toxicity in rats. Data are expressed as mean ± SEM (n = 6). Columns with dissimilar letters are significantly different at p < 0.05.

A remarkable disturbance in BTB has been noticed in ACR-administered group as indicated by elevation of testicular MMP-9 level and diminishing in testicular CX-43 content, when compared to the control rats. It is worth noting that eugenol treatment at dose 100 mg/kg bw succeeded to normalize testicular MMP-9 and CX-43 levels (Fig. 4a and b, respectively).

#### Effect of eugenol treatment on testicular autophagy and apoptotic markers

As depicted in Fig. 4c and d, a significant activation of autophagy and apoptosis process in testicular tissue of ACR-group has been exhibited as evidenced by boosting in testicular Beclin1 and caspase-3 levels, compared to the normal rats. However, oral administration of eugenol resulted in controlling in autophagy and apoptosis process in testicular tissue by reducing testicular Beclin 1 and caspase-3 levels in a dose dependent manner, compared to ACR-intoxicated rats. By further inspection of the results, it can be inferred the superiority of eugenol at high dose in normalization of testicular Beclin 1 and caspase-3 levels.

#### Effect of eugenol treatment on steroidogenic enzymes

**Figure 5:**
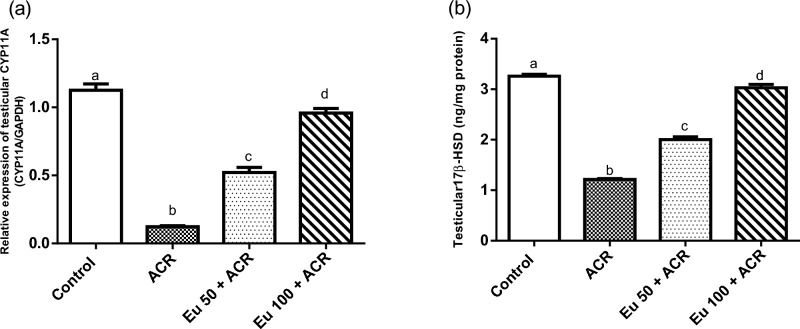
Effect of eugenol treatment on steroidogenic enzymes; CYP11A1 mRNA (**a**), and 17HSD (**b**) levels in testicular tissue in ACR-induced testicular toxicity in rats. Data are expressed as mean ± SEM (n = 6). Columns with dissimilar letters are significantly different at p < 0.05.

An obvious reduction in the levels of steroidogenic enzymes; CYP11A gene expression and 17β-HSD3 was documented in testicular tissue of ACR-administered rats in comparison to the normal control animals. In contrast, oral administration of eugenol at the two dose regimens significantly mitigated the loss of these steroidogenic enzymes levels in testicular tissues (Fig. 5).

### Histopathological findings

#### Effect of eugenol treatment on testicular tissue histopathological alterations

**Figure 6:**
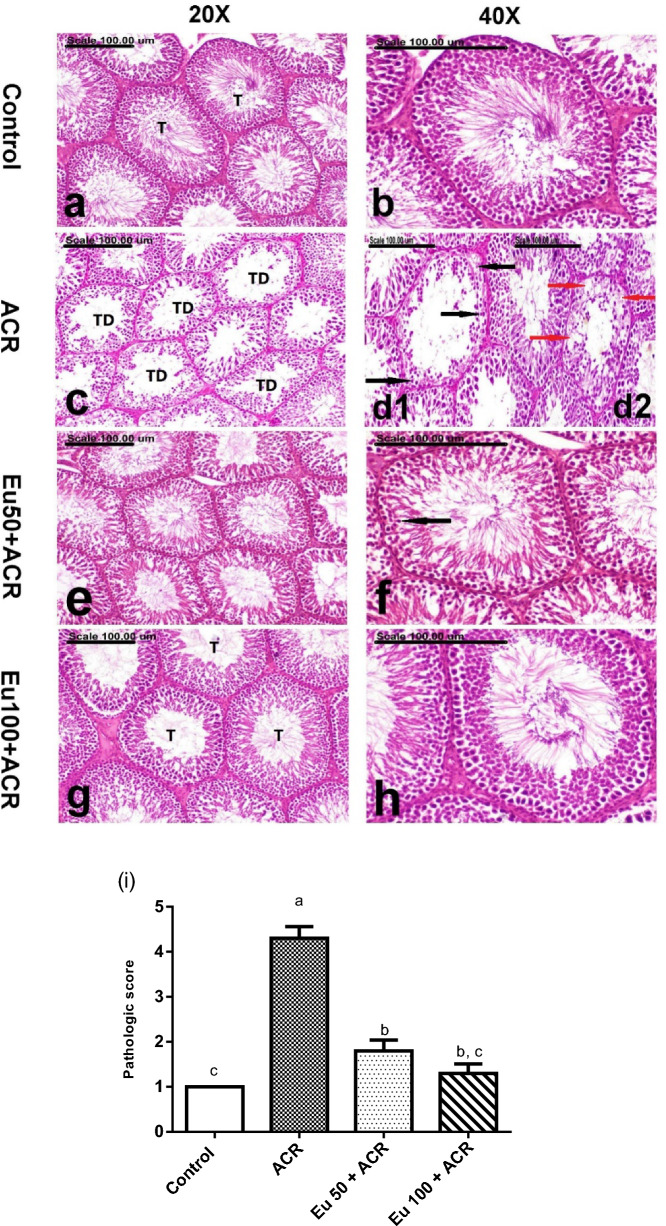
Photomicrograph showing the testes of (**a**,**b**) the control group showing normal seminiferous tubules (T) (**a**) with well-organized spermatogenic series (**b**), (**c**,**d1**,**d2**) ACR group showing progressive testicular degeneration (TD) (**c**) with marked apoptosis of the germ cells, particularly spermatogonia and spermatocytes (black arrows) (**d1**) and extensive vacuolation of Sertoli cells (red arrows) (**d2**), (**e**,**f**) Eu50 + ACR group showing a reduced number of degenerated seminiferous tubules (**e**) and sparse degenerated spermatocytes (black arrow), and (**g**,**h**) Eu100 + ACR group showing normal seminiferous tubules (T) (**g**) with restored spermatogenesis (**h**). (Stain: H&E, scale bar = 100µm). (**i**) The pathologic score of testicular injury recorded in the testes of the control and other treated groups. Data are expressed as mean ± SEM. Values with dissimilar letters are significantly different at p < 0.05.

The testes of the control group revealed normal seminiferous tubules with well-organized spermatogenic series (Fig. 6a and b). Meanwhile, progressive testicular degeneration with marked apoptosis of the germ cells, particularly spermatogonia and spermatocytes, in addition to depletion of round and elongating spermatids, were demonstrated in the testes of the ACR group (Fig. 6c). The degenerated seminefrous tubules are lined by extensively vacuolated Sertoli cells (Fig. 6d). Other frequently demonstrated lesions were the presence of eosinophilic globular bodies and round spermatid giant cells. Significant amelioration, with restored spermatogenesis, was recorded in the Eu50 + ACR and Eu100 + ACR groups. The number of degenerated seminiferous tubules was greatly reduced in the testes of Eu50 + ACR, and sparse degenerated spermatocytes were demonstrated (Fig. 6e and f). Normal seminiferous tubules with restored spermatogenesis were demonstrated in the testes of the Eu50 + ACR group (Fig. 6g and h). The pathologic score of testicular injury recorded in the testes of the control and other treated groups is illustrated in Fig. 6i.

### Immunohistochemistry

#### Effect of eugenol treatment on testicular caspase-3 expression

**Figure 7:**
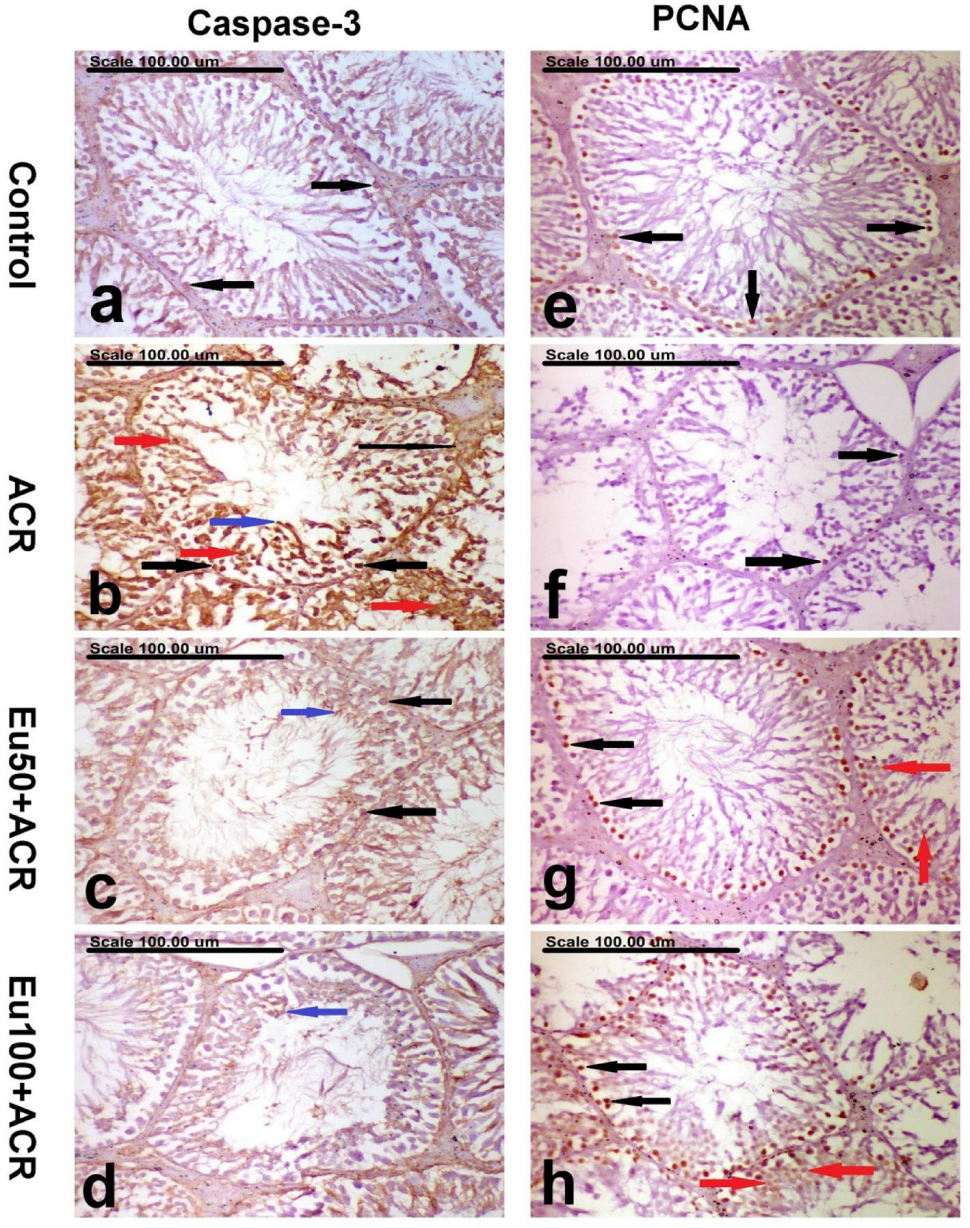
Photomicrograph showing immunohistochemically stained testes of (**a**,**e**) the control group showing sporadic caspase-3-positively stained spermatogonial cells (black arrows) (**a**) and increased PCNA-positively stained spermatogonial cells (black arrows) (**e**), (**b**,**f**) ACR group showing a significant increase in the percentage of caspase-3-positive cells with strong brown perinuclear and/or nuclear staining in the spermatogonium (thick black arrow), spermatocytes (red arrows), spermatids (blue arrow), and leydig cell (thin black arrow), (**b**) and a significant decrease in PCNA-positively stained spermatogonial cells (black arrows) cells (**f**), (**c**,**g**) Eu50 + ACR group showing a significantly decreased percentage of caspase-3-positively stained spermatogonial cells (black arrows) and elongating spermatid (blue arrow) (**c**) and a significant increase of PCNA-positively stained spermatogonial cells (black arrows) and spermatocytes (red arrows) (**g**), (**d**,**h**) Eu100 + ACR group showing sparse caspase-3-positively stained spermatid (blue arrow) (**d**) and a significant increase of PCNA-positively stained spermatogonial cells (black arrows) and spermatocytes (red arrows) (**h**). (Stain: anti-Caspase-3 (**a**–**d**) and anti-PCNA (**e**–**h**), scale bar = 100µm).

The caspase-3 expression results in the testes of the control and other treated groups are illustrated in Table 5. Sporadic caspase-3-positive cells were demonstrated in some seminiferous tubules of the control group (Fig. 7a). A significant increase in the percentage of caspase-3-positive cells with strong brown perinuclear and/or nuclear staining in the spermatogonium, spermatocytes, spermatids, and Leydig cells (Fig. 7b). In comparison to the ACR group, the percentage of caspase-3-positive cells was significantly decreased in the testes of the Eu50 + ACR and Eu100 + ACR, with a significant difference between them (Fig. 7c and d, respectively).

**Table 5:** Effect of eugenol treatment on caspase-3 and PCNA expression in the testes of the control and other treated groups.

Groups	Casapase-3 expression (% of positive cells/HPF) (mean ± SE)	PCNA expression (% of positive cells/HPF) (mean ± SE)
Control	0.30^d^ ± 0.15	2.70^a^ ± 0.21
ACR	3.50^a^ ± 0.22	0.90^c^ ± 0.17
Eu50 + ACR	1.70^b^ ± 0.26	1.90^b^ ± 0.27
Eu100 + ACR	1.10^c^ ± 0.17	2.50^a,b^ ± 0.16

Values with dissimilar letters are significantly different at p < 0.05.

#### Effect of eugenol treatment on testicular PCNA expression

The results of PCNA expression in the testes of the control and other treated groups are illustrated in Table 5. The testes of the control group revealed increased PCNA-positive cells in the seminiferous tubules (Fig. 7e). On the contrary, a marked decrease of PCNA-positive cells was recorded in the testes of the ACR group (Fig. 7f). The immune reaction was mainly demonstrated in the spermatogonium and spermatocytes. A significant increase of PCNA-positive cells was demonstrated in the testes of the Eu50 + ACR and Eu100 + ACR (Fig. 7g and h, respectively).

## Discussion

Significant attention has been directed towards ACR due to its widespread formation during high-temperature processing of foods and its versatile applications across various industries^42^. Accumulative evidences inferred the toxic effects of ACR on male reproductive system including disruption of hormonal status and spermatogenesis, testicular atrophy resulted in infertility^11,12,43^. In the light of this, the present study has been established to investigate the ameliorative effect of eugenol against ACR-induced testicular toxicity. To clarify the mechanism of eugenol in alleviating toxic effects of ACR in male reproductive organ, we performed an in vivo model using male Wistar rats. This research reveals for the protective role of eugenol against ACR-induced testicular toxicity and spermatogenesis dysfunction via modulation of oxidative stress-mediated autophagy and apoptosis associated with AMPK/p-AKT/mTOR signaling pathways, and BTB remodeling/reconstituting.

Prior studies on male rodents reported that ACR toxicity lead to serious testicular insults including swelling, necrosis, vacuolization of spermatids, and irregular multinucleated giant cells formation in seminiferous tubules resulted in its atrophy and apoptosis along with sperm chromosomal aberration, poor sperm viability and number subsequently lead to spermatogenesis impairment^44^. In the present study, ACR exposure was accompanied with marked alteration in sperm characteristics including decline of sperm count, sperm motility and viability along with increasing abnormal sperm number. In addition, a remarkable reduction in the relative testes or epididymides weights was observed following ACR intoxication that may participate in decrease of fluid of seminiferous tubules and androgen content. These detrimental effects of ACR on epididymal sperm count and quality are mainly a consequence of epithelial cells deterioration of the seminiferous tubules and the inhibitory action of ACR on kinesin and dynein cytoskeletal motor protein of sperm flagella resulting in impaired sperm motility^45^.

Evidence has emerged from previous studies postulated that such toxicity of ACR may be attributed to the alkylating action of its metabolite; GA to nuclear spermatid protamines causing dominant lethality of sperms^46,47^. In addition, ACR per se or its metabolite disordered the microtubule motor kinesin protein; responsible for chromosomal segregation and spindle development during cell divisions resulting in decline of sperm maturation and number^48^.

Our data verified a prominent decrease in seminal plasma essential and non-essential amino acids contents following ACRintoxication supporting the observed impairment in sperm count and quality. Another plausible mechanism explained the degenerative effects accompanied ACR exposure on spermatogenesis could be referred to its endocrine effects disrupting steroidogenic hormonal production. In accordance with previous studies, a marked disruption in serum sex hormonal profile/status related spermatogenesis was observed in ACRintoxicated rats indicated by a significant decline in serum testosterone, LH and FSHlevels coupled with repression in testicular steroidogenic enzymes; CYP11A gene expression and 17β-HSD3 levels supporting the endocrine disrupter action of ACR^11,12^. This dramatic decrease in serum sex hormones in ACR-treated rats may be attributed to disorder in the Leydig cells viability and steroidogenic pathway which in turns impairs spermatogenesis^44^. Testosterone plays a main role in sperm production and proper male reproductive function. The biosynthesis of testosterone initiates along with the binding of LH hormone to its receptor on the Leyding cells where testosterone synthesized through signaling cascade effect required CYP11A and 17β-HSD3 as initial and final enzymatic step respectively^49^. Thus the toxic effects of ACR on Leyding cells resulted in its death with a consequence of impaired testosterone biosynthesis.

In contrast, the co-treatment of eugenol at doses 50 and 100 mg/kg for four weeks effectively counteracted these deleterious effects of ACR on sperm count and quality as evidenced by a significant improvement in sperm viability, motility and number with decrease in abnormal sperms. Allied to recent study that accomplished the protective effect of eugenol on sperm parameters mediated through its antioxidant capacity thus persevering spermatogenic cells density and spermatozoa number^50^. Also, eugenol treatment successfully maintained the level of essential and non-essential amino acids in seminal plasma fluid along with restored the serum levels of testosterone, LH and FSH hormones, and testicular stroidogenic enzymes; CYP11A and 17β-HSD3 as well as the relative testes or epididymides weights endorsing its protective role against ACR induced testicular dysfunction in rats.

Collecting multiple evidences highlighted oxidative stress incidence as a principle hallmark of ACR toxicity pathogenesis. ACR targets GSH stores and forming GSH-S-conjugates thus triggering intracellular electrophiles metabolism^51^. Moreover, oxidative stress was found to be highly linked to spermatogenesis defects through liberating lipid peroxides that disrupts the lipid matrix integrity of spermatozoa membrane causing a prompt depletion of intracellular ATP that reduced sperm motility and sperm viability, raised number of abnormal sperms and consequently spermatogenesis dysfunction. Additionally, ROS triggers noteworthy oxidative damage to the sperm organelles via destruction of lipids, proteins and DNA, resulting in sperm death^52^.

In this context, our results indicated that ACR intoxication was associated with boosting in MDA and NO levels as well as reduction in GSH, oxidized and reduced form, content in the testicular tissue confirming the incidence of oxidative stress. These findings coincide with prior study^12^. These deleterious effects of ACR were accompanied by significant changes in testicular energy cell as demonstrated by increasing ATP depletion, AMP/ATP ratio and reduction in AEC resulted in a remarkable up-regulation in testicular AMPK gene expression compared to the control group.

The present study revealed that eugenol treatment dose dependently restored testicular ATP content, AMP/ATP ratio and AEC thus controlled the hyper-activation of testicular AMPK gene expression. This could be due to its antioxidant effects as manifested by increasing GSH (reduced and oxidized form) and lessened MDA and NO contents in testicular tissue, which agrees with the results shown by Ekinci Akdemir et al.^50^. In addition, eugenol administration to ACR intoxicated animals effectively preserved the testicular membrane structure through maintaining its PS and PC content. All these findings supported the positive role of eugenol in maintaining spermatogenesis.

Former studies have reported the empirical role of PI3K/p-Akt/mTOR pathways in testicular function^53,54^. A significant down regulation in PI3K/p-Akt/ mTOR signaling pathway has been observed in the current study upon ACR intoxication that coincides with previous results^12^. In addition, ACR perturbed the BTB construction as inferred by the observed imbalance between the testicular MMP-9 and CX-43 protein content that similar to prior study^12^.

PI3K is most likely to have a signal cascade effect in regulating multiple cell process including apoptosis, autophagy and cellular growth via activating AKT through phosphorylation. Upon activation of AKT, the metabolic mTOR pathway has been stimulated^55^. Recently, a great attention has been paid for mTOR pathway due to its vital role in regulating various essential cellular processes including spermatogenesis. Several studies confirmed the central role of mTOR in maintaining and proliferation spermatogonial stem cell (SSC) and SCs, as well as in BTB rearrangement during spermatogenesis^56,57^. Structurally, mTOR exists in two isoforms, mTORC1 and mTORC2 complex that involved in regulating various biological processes, viz*.,* autophagy, apoptosis, and spermatogenesis in mammalians^58^.

Interestingly, these two complexes have distinct roles in remodeling BTB during spermatogenesis. The mTORC1 is responsible for relaxing BTB through stimulating the production of MMP-9 from SCs leading to destruction of protein adhesion molecules of BTB consequently relax it. On the other hand, mTORC2 has been elicited for preserving the dense/compact structure of BTB by producing CX-43 which is responsible for retightening BTB through restoring the communication between the tight junction complexes and gap junction. This arrangement of BTB enables the spermatocytes to migrate to the adluminal part throughout preleptoten phase where meiosis division occurs during spermatogenesis process^59^.

Another issue of interest is mTOR signaling involvement in regulating autophagy and apoptosis processes through the inhibitory action of mTORC1 on these pathways. Under oxidative stress conditions, mTOR pathway has been negatively regulated thus triggered cellular autophagy and apoptosis which have been found to be linked each other. Where, under certain conditions, autophagy has been shown to promote autophagic cell death by overly destroying the cytoplasm^60^. Similar to the literature, it was observed that ACR increased Beclin-1 production; autophagy marker and caspase-3; apoptotic indicator activation in testicular tissue^12^. According to Tian et al. under oxidative stress burst, aberrant autophagy was observed in testicular tissue^61^. Furthermore, the energy-sensor AMPK was found to be participated in induction of cell autophagy by either directly activating ATG1 or by blocking the downstream PI3K/Akt/mTOR signaling cascade^62–64^.

By contrast, eugenol treatment suppressed testicular Beclin-1 and caspase-3 content exerting anti-autophagy and anti-apoptotic effects. That is highly related to its antioxidant properties and the positive regulatory role of eugenol on mTOR pathway as indicated by a significant upregulation of testicular PI3K/p-Akt/mTOR mechanisms, compared to ACR group. Moreover, eugenol administration successfully preserved the BTB compact structure through regulating MMP-9 and CX-43 levels in testicular tissue.

Additional evidence supporting the incidence of oxidative stress as a consequence of ACR exposure is the significant decline in nuclear content of Nrf2 level in testicular tissue compared to the control group. Activation of Nrf2-regulated antioxidant signaling pathways is an important target for alleviating ROS-provoked cellular damage and maximizing antioxidant defense mechanism^65^. Beside, its significant role in mediating cell survival and promoting cell growth pathway, AKT was informed to induce nuclear localization of Nrf2^66^. Hence, production of an array of antioxidant and detoxifying enzymes is up-regulated^67^.

Our results documented that eugenol effectively ameliorated the ACR-provoked ROS-overproduction via augmented testicular Nrf2 nuclear translocation through enhancing phosphorylation of AKT, in respect to the ACR-challenged group. Additionally, ROS production is involved in apoptosis incidence via triggering DNA fragmentation, and endogenous mitochondrial damage through disrupting electron transport chain and in consequence impairs regulation of energy^68,69^.

The histopathological and immunohistochemical analyses also proved the detrimental effects of ACR on rat testicular tissue and confirmed the biochemical findings. Similar to the literature, ACR induced progressive testicular atrophy with marked apoptosis of the germ cells, degeneration of seminiferous tubules and vacuolization of SCs^11^. Meanwhile, eugenol treatment effectively alleviates the testicular histopathological alterations and restores spermatogenesis as well as mediated caspase-3 and PCNA protein expression thus regulating testicular apoptosis and proliferation compared to ACR group.

## Conclusion

**Figure 8:**
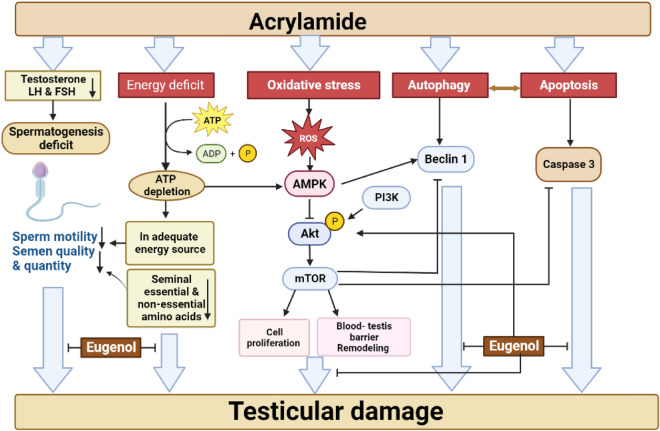
An illustrative figure showing the possible mechanisms of action of eugenol in ameliorating the toxic effects of acrylamide on testicular tissue.

Collectively, the current study offers compelling evidence regarding the gonadoprotective effects of eugenol against the degenerative impacts on the male reproductive system induced by ACR exposure. Eugenol achieves this protection by preserving the integrity and function of testicular tissue through the mediation of AMPK/p-AKT/mTOR signaling pathways, maintaining BTB integrity, and exhibiting anti-autophagy and anti-apoptotic actions, as illustrated in Fig. 8. Given these findings, we recommend further investigations to delve into the molecular mechanisms that further elucidate the potential role of eugenol in male reproductive organ health.

## Data Availability

All data generated or analyzed during this study are included in this published article.
